# Apremilast is a potentially useful treatment for severe palmoplantar pustulosis with extra‐palmoplantar symptoms

**DOI:** 10.1002/ski2.336

**Published:** 2024-01-15

**Authors:** Ayano Fukushima‐Nomura, Saeko Takamiyagi, Risa Kakuta, Yoshihiro Ito, Ikuko Hirai, Junichi Umemoto, Hironari Hanaoka, Yuko Kaneko, Keiji Tanese

**Affiliations:** ^1^ Department of Dermatology Keio University School of Medicine Tokyo Japan; ^2^ Horinouchi‐ekimae Dermatology Clinic Yokohama Japan; ^3^ Department of Rheumatology Keio University School of Medicine Tokyo Japan

## Abstract

Palmoplantar pustulosis (PPP) is a chronic inflammatory skin disorder affecting the palms and soles. In rare cases, severe patients develop acute extra‐palmoplantar lesions often accompanied by arthralgia. Such cases with extensive symptoms often necessitate systemic treatments with variable efficacy and potential side effects. Apremilast, known for its broad immune response modulation, presents promise as a therapeutic option for severe PPP with joint and extra‐palmoplantar lesions. This case highlights apremilast as a potential systemic treatment for such cases with minimal side effects.

## Graphical abstract

Rare cases of severe palmoplantar pustulosis with rapidly developing extra‐palmoplantar lesions and joint pain have been treated by immunosuppressants with variable efficacy and side effects from prolonged treatment. Our case was successfully treated with apremilast, which suggests apremilast as a potential systemic treatment for such cases.

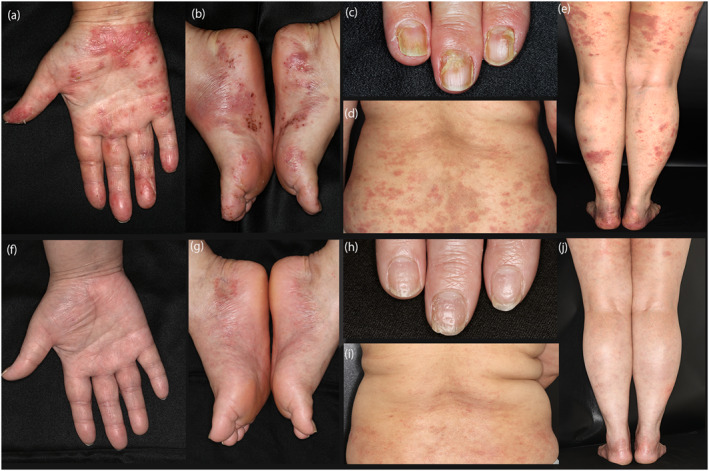

## 1 INTRODUCTION

Apremilast, an oral phosphodiesterase‐4 inhibitor, modulates diverse immune dysregulations. It is currently approved for psoriasis, psoriatic arthritis, and oral ulcers in Behçet's disease in Japan and is also expected to be useful in other chronic inflammatory diseases.^1^ Here, we report its potential efficacy on severe palmoplantar pustulosis (PPP) with extra‐palmoplantar skin lesions and synovitis.

## 2 CASE REPORT

**FIGURE 1:**
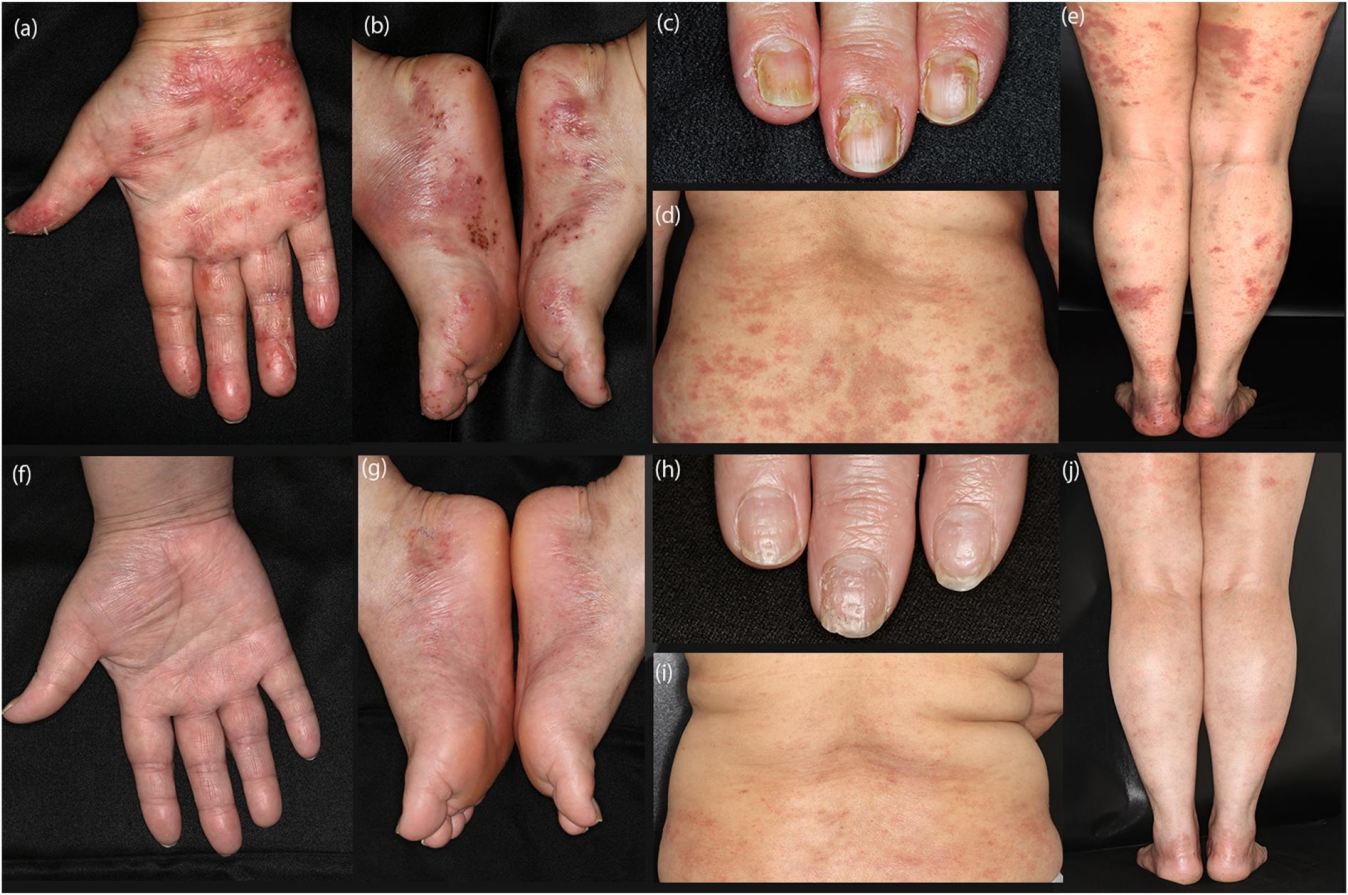
Clinical manifestations (a–e) before and (f–j) 1 year after treatment with apremilast. (a,b) Large, painful pustules repeatedly developed in the palms and soles. (c) Nail deformities in the proximal areas of the nails. Note the oedematous erythema in the posterior nail fold. (d,e) Extensive skin lesions in extra‐palmoplantar regions. Poorly demarcated erythema with papules and pustules on the trunk. (f,g) Skin lesions on the palms and soles showed marked improvement with apremilast. (h) The erythema in the posterior nail fold and the disruptive nail deformity are resolved. Small pittings reside in some of the nails. (i,j) Improvement of skin lesions in the trunk and limbs with light pigmentation.

**FIGURE 2:**
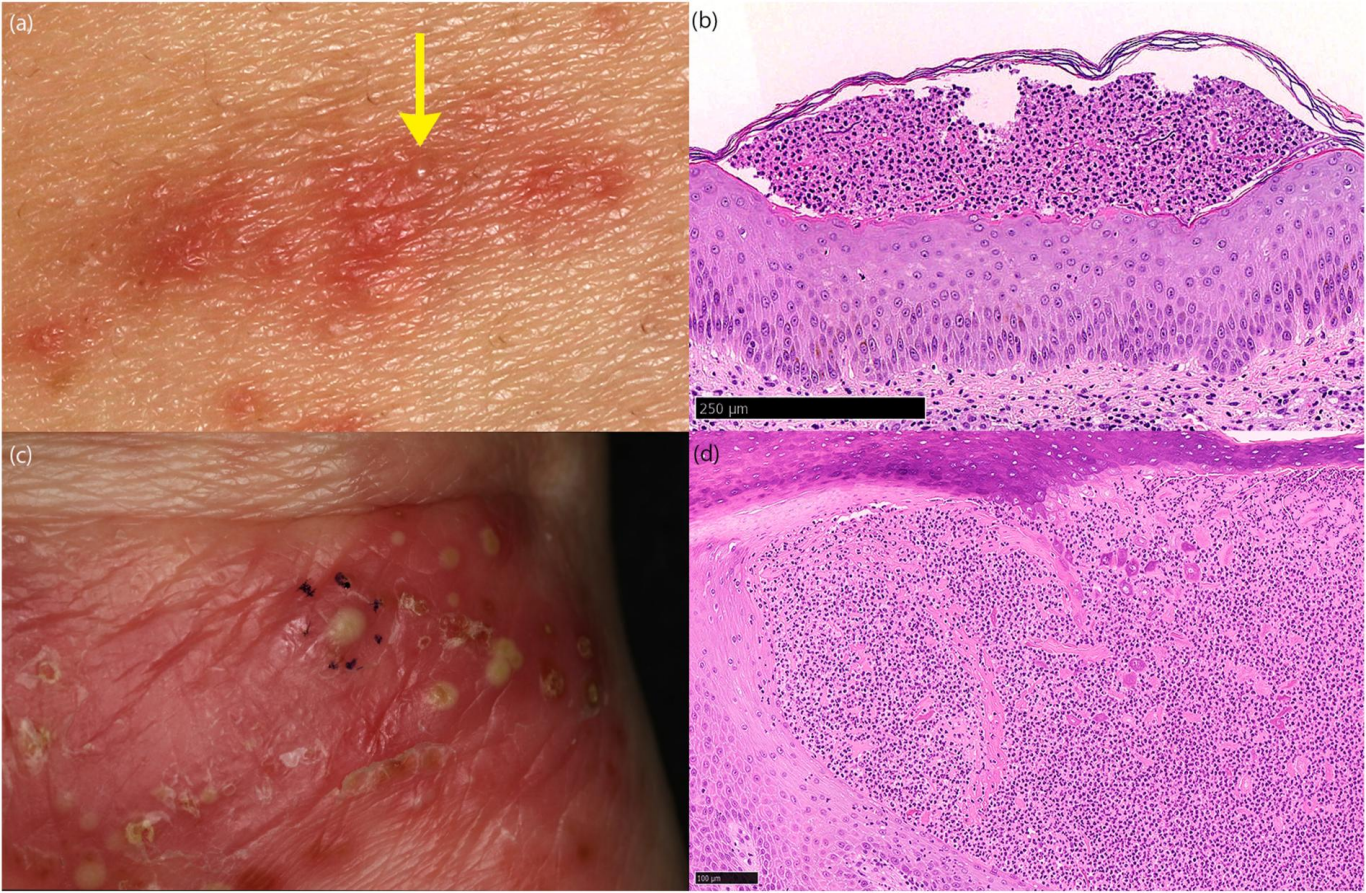
(a) Close‐up image of extra‐palmoplantar skin lesions. Poorly demarcated erythema with papules and pustules on the trunk (yellow arrow). (b) Histopathology of the skin specimen taken from the extra‐palmoplantar skin lesion in the trunk showed mild acanthosis and subcorneal accumulation of neutrophils (haematoxylin–eosin staining, scale bar = 250 μm). (c) Close‐up image of a lesion on the heels, close to the sole. Skin specimen was taken from the pustule circled in black dots. (d) The specimen showed subcorneal accumulation of neutrophils (haematoxylin–eosin staining, scale bar = 100 μm).

A 41‐year‐old, non‐smoker woman was diagnosed clinically as PPP for eruptions on her hand and sole. Three months later, she developed eruptions on her trunk and extremities and was referred to our hospital. Clinically, scaly erythema with large pustules were noted on the palms and soles, and irregularly shaped erythema with thin scales and sterile pustules were distributed on the trunk and extremities (Figure 1, 2). In addition, all fingernails were deformed, and pain and stiffness developed in the left third finger's distal interphalangeal joint. Ultrasound examination of the affected joint revealed enthesitis, and bone scintigraphy showed no signs of other affected joints. Histopathologically, biopsy specimens of the trunk and sole showed subcorneal neutrophilic micropustules (Figure 2b,d). From these findings and the lack of systemic symptoms such as fever or malaise, she was diagnosed as PPP with extra‐palmoplantar legion and pustulotic arthro‐osteitis (PAO). Screening for focal infections revealed chronic sinusitis, which was treated with antibiotics with no effect. As the skin lesions were refractory to topical corticosteroids and vitamin D3 treatments and the arthralgia was progressive, she was started on apremilast. Subsequently, palmoplantar lesions and arthralgia almost completely resolved after 3 months, with improved nail deformity. The extra‐palmoplantar skin lesions also improved; and were further resolved with additional 6 months of Narrowband Ultraviolet B phototherapy, with a total cumulative dose of 17 J/cm^2^ (Figure 1f–j). She did not report any adverse events due to apremilast.

## 3 DISCUSSION

Palmoplantar pustulosis is a chronic inflammatory skin disorder affecting the palms and soles. In rare cases, severe patients with PPP experience the rapid emergence of extra‐palmoplantar lesions characterised by scaly erythema and pustules, often accompanied by joint pain. Skin manifestations may resemble pustular psoriasis; however, a key distinguishing feature of PPP is the absence of systemic symptoms, such as high fever, chills, and systemic inflammation.^2^ Treatment of PPP includes phototherapy, topical corticosteroids, and vitamin D analogues. In patients with focal infections or smoking habits, resolving these issues may be beneficial. Severe cases with extensive symptoms, including extra‐palmoplantar skin lesions, nail deformities, and PAO, sometimes require systemic treatments such as oral retinoids, methotrexate, and cyclosporine.^3^ However, their efficacy is variable, and patients suffer side effects from prolonged therapies.^1^, ^4^

Recently, guselkumab, an anti‐IL‐23p19 monoclonal antibody, has been approved for PPP in Japan. While both apremilast and guselkumab have been shown to be beneficial for PAO,^4^, ^5^ the efficacy of guselkumab for skin lesions may not be as compelling as apremilast; only approximately half of the patients achieved 50% improvement by guselkumab, whereas 78.3% of patients on apremilast achieved the same level of improvement in clinical trials.^6^, ^7^, ^8^ In addition, a sub‐analysis by the Pharmaceuticals and Medical Devices Agency, the drug regulatory authority of Japan, suggested that the effectiveness of guselkumab in nonsmokers may be low.^9^ Furthermore, biologics including guselkumab, are generally more economically burdensome than apremilast.^10^

**FIGURE 3:**
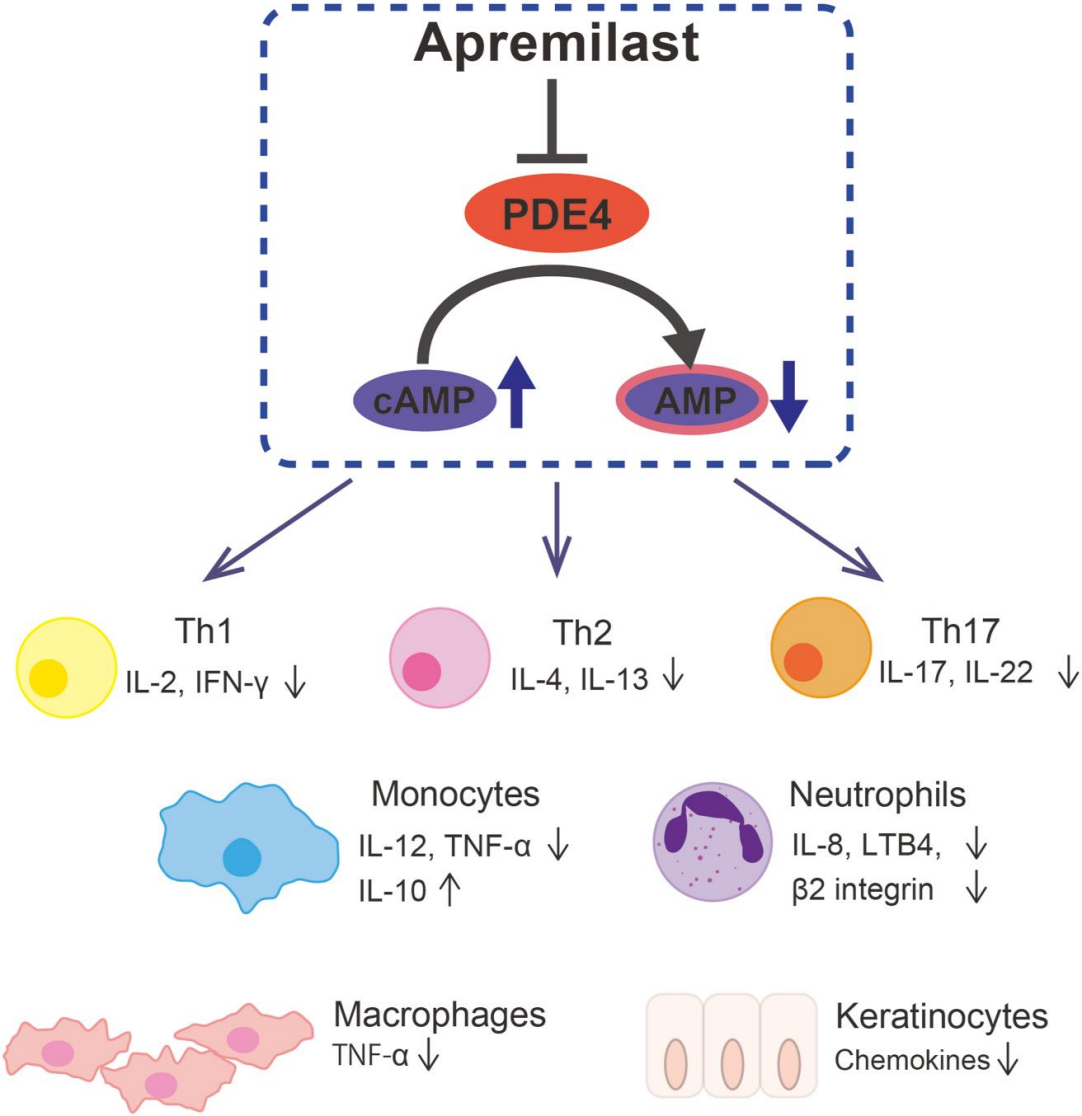
A diagram showing the mode of action of apremilast in Palmoplantar pustulosis (PPP), based on the proposed mechanism of action in various cell types observed in vitro studies. Apremilast modulates a broad spectrum of inflammation via PDE4 inhibition in diverse immune cells and tissue‐specific cells in the skin and joints. IL, interleukin; LTB4, leukotriene B4; TNF, tumour necrosis factor.

In our case, considering the patient's financial burden and based on the pathological proximity of PPP and psoriasis, apremilast was administered before guselkumab, which significantly improved both the skin lesions and PAO. Although PPP and psoriasis share activation of the IL‐23/IL‐17 immune pathway, a recent single‐cell study has shown that type 2 inflammation is also upregulated in PPP,^11^ suggesting that treatment of PPP requires the inhibition of various inflammatory pathways. Apremilast may be a beneficial therapeutic option because it broadly modulates these immune responses and neutrophil activation, another pathophysiological hallmark of PPP (Figure 3).^1^, ^12^ Our case suggests that apremilast is a promising systemic drug for severe PPP with extensive symptoms in the joint and skin, warranting further case accumulation.

## CONFLICT OF INTEREST STATEMENT

The authors declare no conflicts of interest.

## AUTHOR CONTRIBUTIONS

**Ayano Fukushima‐Nomura**: Investigation (lead); Writing – original draft (lead). **Saeko Takamiyagi**: Writing – review & editing (supporting). **Risa Kakuta**: Writing – review & editing (supporting). **Yoshihiro Ito**: Writing – review & editing (supporting). **Ikuko Hirai**: Writing – review & editing (supporting). **Junichi Umemoto**: Writing – review & editing (supporting). **Hironari Hanaoka**: Writing – review & editing (supporting). **Yuko Kaneko**: Writing – review & editing (supporting). **Keiji Tanese**: Writing – review & editing (supporting).

## ETHICS STATEMENT

The patient provided informed consent for the publication of this report.

## Data Availability

Data sharing is not applicable to this article as no new data were created or analyzed in this study.
